# Comparative Analysis of Muscle Nutrients, Lipid Metabolism and Intestinal Microbiota in *Pelteobagrus fulvidraco* from Rice Field and Pond Culture Modes

**DOI:** 10.3390/foods15152666

**Published:** 2026-07-29

**Authors:** Linjun Zhou, Rui Jia, Yiran Hou, Chengfeng Zhang, Bing Li, Jian Zhu

**Affiliations:** 1 Key Laboratory of Integrated Rice-Fish Farming Ecology, Ministry of Agriculture and Rural Affairs, Freshwater Fisheries Research Center, Chinese Academy of Fishery Sciences, Wuxi 214081, China; zhoulinjun@ffrc.cn (L.Z.);; 2 Wuxi Fisheries College, Nanjing Agricultural University, Wuxi 214081, China

**Keywords:** integrated rice–fish farming, muscle nutrients, metabolic profile, intestinal microbiota, *Pelteobagrus fulvidraco*

## Abstract

Integrated rice–fish farming is increasingly promoted as a sustainable aquaculture model, yet its effects on flesh quality and gut microbial assembly in *Pelteobagrus fulvidraco* remain insufficiently understood. Here, we compared pond-cultured (PP) and rice-field-cultured (PR) individuals using growth measurements, muscle amino acid and fatty acid profiling, untargeted LC-MS/MS metabolomics and 16S rRNA gene sequencing. Body weight and length did not differ significantly between groups, whereas PP fish showed greater body width and height. PR fish contained higher levels of Ser, Val, Ile and total essential amino acids, together with amino acid and energy-related metabolites. In contrast, PP fish showed higher concentrations of saturated fatty acids, monounsaturated fatty acids, n-3 polyunsaturated fatty acids, total fatty acids and lipid-associated metabolites. The gut microbiota also differed markedly between culture systems. PP fish exhibited higher α-diversity and enrichment of Proteobacteria, Firmicutes, *Pseudomonas* and *Ralstonia*, whereas PR fish were characterized by Verrucomicrobiota, Fusobacteriota, *Akkermansia* and *Cetobacterium*. Predicted functions suggested enhanced carbohydrate and energy metabolism in PR fish and stronger environmental-response-related functions in PP fish. These findings indicate that pond and rice-field culture promote distinct quality-forming pathways in yellow catfish, providing a basis for optimizing rice–yellow catfish co-culture toward quality-oriented production.

## 1. Introduction

As global aquaculture moves towards greener and more sustainable production, integrated rice–fish farming has attracted increasing attention as an ecologically based aquaculture model that combines grain production, aquatic animal supply, nutrient recycling and environmental improvement [1,2,3]. Compared with conventional pond culture, rice paddy aquaculture provides a more heterogeneous habitat, in which rice-canopy shading, modified water physicochemistry, proliferation of natural food organisms and recycling of aquaculture wastes can jointly reshape the rearing environment and thereby affect fish growth, nutrient deposition and health status [4,5]. In recent years, increasing attention has been paid to how farming systems determine aquatic product quality. Studies have shown that rice–fish co-culture can alter muscle proximate composition, amino acid profiles, fatty acid composition and flesh-quality traits. Consistent with this, rice-field cultured tilapia have been reported to contain higher muscle levels of total umami amino acids and monounsaturated fatty acids than tank-reared individuals [6]. Another study found that rice-field-cultured crucian carp exhibited significantly higher levels of amylase, trypsin and several antioxidant enzymes than pond-cultured individuals [5]. The enriched natural food web and generally lower stocking intensity in paddy ecosystems may also increase fish activity and diversify nutrient intake, thereby favoring protein deposition and physiological fitness [7]. Beyond muscle nutrition, rice-field-culture has been associated with shifts in intestinal microbial diversity and the enrichment of bacterial taxa involved in nutrient utilization, immune regulation and environmental adaptation [6,8]. With advances in high-throughput sequencing and metabolomics, accumulating evidence further indicates that rearing environments can modulate host nutritional metabolism and flesh-quality formation through gut microbial pathways and their interactions with host metabolic networks [8,9].

Nevertheless, available evidence is not fully consistent across species and farming systems. In some contexts, conventional pond culture may promote greater lipid deposition than rice-field culture because of its more predictable supply of formulated feed and higher dietary energy input, resulting in higher crude lipid levels or increased concentrations of total and selected fatty acids in muscle [10]. A study on *Opsariichthys bidens* showed that rice-field-cultured individuals had significantly lower muscle lipid content than pond-cultured fish; in contrast, pond-cultured individuals exhibited higher levels of unsaturated fatty acids, total ω-3 fatty acids and total ω-6 fatty acids than those cultured in rice fields [11]. Another study similarly reported that tilapia cultured in rice-field systems had higher muscle protein content and lower lipid content, a pattern partly attributed to improved water quality and greater swimming activity in the rice-field environment [12].

Conversely, several studies have shown that rice-field culture can also enhance the accumulation of specific muscle fatty acids, particularly nutritionally important unsaturated fatty acids. A study in tilapia found that rice–fish co-culture significantly increased muscle docosahexaenoic acid (DHA) compared with conventional culture and this increase was potentially associated with shifts in intestinal microbiota [13]. Similar patterns have been observed in crucian carp, where rice–fish farming increased several individual fatty acids, including C16:0, C17:0, C16:1n7, C20:3n3, arachidonic acid, eicosapentaenoic acid and DHA, and resulted in a higher n-3 PUFA fraction relative to pond-cultured fish [5]. Therefore, evaluating farming systems solely on the basis of growth performance or a single nutritional endpoint is unlikely to capture their full biological effects. An integrative framework combining nutrient composition, fatty acid profiling, metabolomics and intestinal microbiome analysis is required to clarify how rearing environments shape fish flesh quality and host physiological adaptation [14,15].

Yellow catfish (*Pelteobagrus fulvidraco*) is a high-value freshwater aquaculture species in Asia, valued for its rapid growth, delicate flesh and high consumer acceptance [16,17]. Its production in China has expanded rapidly under intensive culture, yet pond-based production can be associated with excessive lipid deposition, off-flavor formation and deterioration of flesh quality [18]. Although yellow catfish has historically been produced mainly in pond systems, it is increasingly being incorporated into integrated rice–fish farming, where fish occupy paddy refuges and interact with rice growth, natural food webs, sediment processes and the paddy-field microbiome [2,18,19]. Previous studies have shown that rice–fish systems can reshape the rearing environment, gut microbiota and host metabolic responses in farmed fish, with potential consequences for muscle nutrition and product quality [20,21]. However, evidence for yellow catfish remains limited, and existing work has focused largely on agricultural performance, environmental effects or microbiota–metabolite changes during specific rice growth stages [2,7]. Whether rice-field culture systematically alters muscle nutrient composition, muscle metabolic phenotype and intestinal microbial assembly in yellow catfish therefore remains unclear. Here, we compared yellow catfish reared in conventional pond culture (PP) and rice-field culture (PR), integrating muscle amino acid and fatty acid profiling, untargeted muscle metabolomics and 16S rRNA-based gut microbiome analysis. We show that rearing mode markedly reshapes the nutritional and physiological phenotype of yellow catfish, with pond- and rice-field-cultured individuals displaying distinct growth traits, muscle-quality signatures and gut microbial configurations. By linking nutrient deposition, metabolic reprogramming and microbial community structure within a single analytical framework, this study provides new insight into flesh-quality formation across contrasting aquaculture systems and offers a basis for optimizing rice–fish co-culture for high-quality yellow catfish production.

## 2. Materials and Methods

### 2.1. Experimental Set-Up and Sample Collection

This study was performed at the Jingjiang Experimental Base, Freshwater Fisheries Research Center, Jingjiang City, Jiangsu Province, China. Three ponds (PP) and three rice–yellow catfish co-culture paddy fields (PR) were involved in this study, and each of them covered an area of approximately 400 m^2^. More narrowly, each paddy consists of two zones, including a rice planting field (0.3 m in depth, 340 m^2^) and a canal refuge (1.2 m in depth, 60 m^2^) [2]. All *P. fulvidraco* were in good health with an initial body weight of 145.3 ± 5.2 g, and the stocking density was set as 187 g/m^2^ in both culture systems. All fish used in this study were obtained from self-bred stocks maintained at the base. We fed the fish daily with the commercial feed (HAID Group Co., Ltd., Guangzhou, China) including 42.0% crude protein, 5.0% crude fat, 3.0% crude fiber, 16.0% crude ash, and 0.80% total phosphorus at approximately 1% of their body weight. During the 90-day trial, the feeding amount was adjusted every 15 days. At each adjustment, representative fish were randomly captured from each pond or rice-field unit, weighed, and then returned to the corresponding culture unit. The updated mean body weight, together with the number of surviving fish after correction for any recorded mortality, was used to estimate the current biomass of each unit. The daily feed ration was then recalculated as approximately 1% of the updated biomass. The same protocol was used for the PP and PR groups. During the trial, the water temperature (22.3 to 33.5 °C), dissolved oxygen (4.4 to 5.0 mg/L), and pH (7.4 to 8.1) were periodically assessed.

Upon termination of the trial, 30 individuals with similar body size were randomly collected from each pond or rice-field replicate and were measured for body weight, body length, body breadth and body height. For amino acid, fatty acid and untargeted metabolomic analyses, muscle tissues from the 30 fish collected from each pond or rice-field replicate were pooled to generate one representative muscle sample. Therefore, three pooled muscle samples were obtained for each culture mode and used as pooled biological replicates for these analyses (*n* = 3 per group). For intestinal microbiota analysis, individuals with similar body size and the same sex were selected from the 90 fish in each treatment group. Intestinal samples from every six fish were randomly pooled to generate one sample, resulting in five pooled intestinal samples per group (*n* = 5 per group). All procedures involving animals were carried out in compliance with animal welfare guidelines and were approved by the Freshwater Fisheries Research Center. Subsequently, the euthanized specimens (via overdose of MS-222) were dissected to harvest the intestinal and skeletal muscle tissues, which were flash-frozen in liquid nitrogen and preserved in −80 °C for subsequent investigations.

### 2.2. Determination of Amino Acids in Muscle

Hydrolyzed and free amino acids were determined using two separate preparation procedures. For hydrolyzed amino acid analysis, approximately 100 mg of wet muscle tissue was mixed with 15 mL of 6 mol/L hydrochloric acid and hydrolyzed at 110 °C for 22 h. After hydrolysis, the solution was filtered, and 1 mL of the filtrate was concentrated under reduced pressure at 40 °C. The dried residue was reconstituted with sodium citrate buffer (pH 2.2), filtered through a 0.22 μm membrane, and analyzed using an automatic amino acid analyzer (Hitachi LA8080, Tokyo, Japan). This procedure was used to quantify acid-hydrolyzed amino acids released from muscle proteins. Tryptophan was not determined because it is degraded during strong acid hydrolysis and was not included in the reported amino acid sums. Other amino acids that may be influenced by acid hydrolysis were compared under the same pretreatment and analytical conditions across all samples.

Free amino acids were analyzed using a separate extraction protocol. Approximately 100 mg of wet muscle was extracted with 0.02 mol/L hydrochloric acid in a 10 mL centrifuge tube. The extract was purified on a C18 solid-phase extraction cartridge preconditioned with methanol and water. After sample loading, the cartridge was eluted with 0.02 mol/L hydrochloric acid, and the eluate was adjusted to 5 mL with the same solution. The final extract was filtered through a 0.45 μm membrane and analyzed using an automatic amino acid analyzer (Hitachi LA8080, Tokyo, Japan). Amino acids were separated on a sulfonic acid cation-exchange resin column and detected at 570 and 440 nm. The injection volume was 20 μL and the reaction temperature was maintained at 135 ± 5 °C.

### 2.3. Test of Fatty Acids in Muscle

Muscle tissue samples (200 mg) underwent acid hydrolysis with 10 mL of 8.3 M hydrochloric acid at 70–80 °C for 40 min. Following that, total lipids were subsequently extracted from the hydrolysate by mixing with 30 mL of a diethyl ether and petroleum ether solution (1:1, *v*/*v*) [10]. For methyl esterification of the extracted lipids, a 14% boron trifluoride-methanol solution was employed at 45 °C for 20 min. The resulting fatty acid methyl esters (FAMEs) were analyzed via gas chromatography (Agilent 7890A, Santa Clara, CA, USA). The injector and detector temperatures were set at 250 °C and 260 °C, respectively. Identification of fatty acid components was achieved by matching retention times against 37 FAME reference standards (Sigma, Laramie, WY, USA).

### 2.4. Untargeted Metabolomic Profiling in Muscle

Tissue samples (100 mg) were homogenized and subsequently suspended in ice-cold 80% methanol. Following centrifugation, the supernatant was diluted to achieve a final methanol concentration of 53%. Diluted solutions were then transferred to new Eppendorf tubes and subjected to centrifugation. The resulting supernatant was directly analyzed using an LC-MS/MS system that was performed using the Vanquish UHPLC system (Thermo Fisher, Germering, Germany) coupled with an Orbitrap Q Exactive HF mass spectrometer (Thermo Fisher, Bremen, Germany) [22]. The generated raw data were processed using Compound Discoverer 3.1 (CD3.1, Thermo Fisher) to conduct quantitation for metabolites. These identified metabolites were annotated using the KEGG, HMDB and LIPIDMaps databases. The package metaX (version 1.4.2) was used to conduct principal components analysis (PCA) and partial least squares discriminant analysis (PLS-DA) for processing metabolomics data. The univariate analysis (*t*-test) was employed to calculate the statistical significance (*p*-value) and metabolites with VIP > 1 and *p*-value < 0.05 were considered to be significantly different.

### 2.5. 16S rRNA Sequencing in Intestinal Bacteria

Intestinal microbial DNA was isolated using the HiPure Soil DNA Kit (Magen, Guangzhou, China). We then chose the V3-V4 region of the ribosomal RNA gene and amplified it with PCR primers 341F (5′-CCTACGGGNGGCWGCAG-3′) and 806R (5′-GGACTACHVGGGTATCTAAT-3′). Amplified fragments were purified with AMPure XP Beads (Axygen, Union City, CA, USA) and were quantified by real-time PCR. After that, the obtained product was sequenced via an Illumina NovaSeq platform. Subsequently, we obtained the clustered effective tags from the reads via the Uparse algorithm and then classified these into OTUs based on a 97% sequence identity criterion [23]. The OTUs were annotated and classified by employing the Mothur method along with the SILVA database [24]. Alpha diversity indices, including Chao1, Shannon and Simpson, were calculated using QIIME (V1.7.0). Beta diversity analysis was conducted with QIIME (V1.9.1) to evaluate sample differences in species complexity. Simper function was performed using the Vegan package in R to quantify the contribution of each classification to the differences between the PP and PR groups. LDA effect size (LEfSe) analysis was also used to determine the statistical and biological differences in bacterial communities between the two groups.

### 2.6. Statistical Analysis

Original data sorting was conducted using Excel software (V2021, Microsoft Corporation, Redmond, WA, USA), with further analysis mainly conducted by SPSS software (V24.0, IBM Corporation, Armonk, NY, USA) and the results are presented as the mean ± standard error (mean ± SEM). All data were checked for the normality of distribution (Shapiro–Wilk test) as well as the homogeneity of variances (Levene test). Otherwise, non-parametric tests were applied, including the Mann–Whitney U test for two-group comparisons. Differences across different groups were evaluated by one-way analysis of variance (ANOVA) and the following *post hoc* comparisons were achieved with the LSD test. Additionally, differences in amino acid and fatty acid composition between two groups were analyzed using a *t*-test. Unless otherwise stated, the level of statistical significance was set at *p* < 0.05.

## 3. Results

### 3.1. Growth Performance of P. fulvidraco

After 90 days of feeding, we measured the body length, weight, width and height of yellow catfish captured from different aquaculture systems. We observed morphological alterations in PP individuals with slightly greater body width and height compared to their PR counterparts (*p* < 0.01, Table S1), whereas no significant differences were detected in body length or weight. This suggests that pond-cultured fish had a more robust and fuller body shape, whereas rice-field-cultured fish tended to exhibit a relatively slimmer or more streamlined morphology.

### 3.2. Amino Acid Composition of P. fulvidraco Muscle Samples

The composition of amino acids in the muscle samples of *P. fulvidraco* from both the PP and PR groups is presented in Table 1. Here, we identified seventeen amino acids, including seven essential amino acids and ten non-essential amino acids. Quantitative analysis revealed elevated levels of Ser, Val, Ile and total EAA in the muscle of rice paddy-dwelling *P. fulvidraco* relative to pond-cultured individuals. In addition, the 15 kinds of free amino acid composition in muscle were also quantified. We found significant decreases in Ala, Val, Ile, delicate amino acids, sweet amino acids and total free amino acid (TFAA) in pond-farming groups (Table S2). These results indicate that *P. fulvidraco* reared in rice paddies could exhibit marked superiority in muscle nutrition qualities compared to pond-cultured fish.

### 3.3. Muscle Fatty Acid Composition of P. fulvidraco

The muscle tissue of *P. fulvidraco* was characterized for fifteen fatty acids, which consisted of three saturated fatty acids (SFAs), four monounsaturated fatty acids (MUFAs), and eight polyunsaturated fatty acids (PUFAs). In particular, individuals cultured in PP exhibited higher concentrations of most fatty acids and greater total fatty acid content in muscle than those in PR, indicating that culture mode markedly influenced lipid accumulation (Table 2). Compared with pond-cultured individuals, saturated fatty acid (C14:0, C16:0 and C18:0; *p* < 0.05), monounsaturated fatty acid (C16:1; *p* < 0.05), part of polyunsaturated fatty acid (C18:2n6c, C20:4n6 and C20:3n3; *p* < 0.05) as well as total fatty acid exhibited a significant decrease in the muscle tissues of *P. fulvidraco* from the rice–fish co-culture system. In addition, several individual PUFA components differed significantly between the PP and PR groups; total PUFA did not reach statistical significance (*p* = 0.077). This may be explained by the relatively large variation and non-significant differences in other PUFA components, such as C20:2, C18:3n3, C20:5n3, C22:6n3 and C20:3n6, which weakened the statistical significance of the aggregated PUFA value.

### 3.4. Metabolic Profiling in P. fulvidraco Muscle Tissues

Quality control (QC) analysis indicated that the sequencing data were credible based on the well-clustered QC samples and displayed clear separation between the PP and PR groups in both positive and negative ion modes (Figure S1). Overall, in *P. fulvidraco* muscle tissue, we identified 398 metabolites in positive ion mode and 208 metabolites in negative ion mode. Referring to the Human Metabolome Database (HMDB), the metabolites were predominantly enriched into three categories, including 83 lipids and lipid-like molecules, 24 organoheterocyclic compounds and 76 organic acids and derivatives (Figure S2A,B). Referring to the LIPID MAPS database, the identified lipid metabolites were mainly classified as 42 fatty acids (FAs) and 66 glycerophospholipids (GP) (Figure S2C,D).

**Figure 1:**
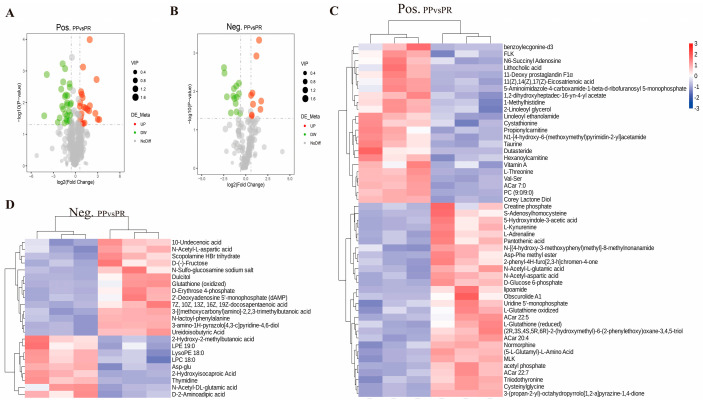
Differential metabolites in the muscle of *P. fulvidraco* between the PP and PR groups. (**A**,**B**) Volcano plot of the differential metabolites in positive (Pos) and negative (Neg) ion modes; (**C**,**D**) Differential metabolites between PP and PR groups in positive (Pos) and negative (Neg) ion modes, respectively. The heatmap displays hierarchical clustering of samples (rows) and metabolites (columns), where shorter branches in the dendrogram correspond to greater similarity. Note: Samples were analyzed in both positive and negative ionization modes to maximize metabolite coverage.

Individuals from PR groups exhibited significant differences in 51 metabolites (up 28, down 23) in positive ion mode and 23 metabolites (up 14, down 9) in negative ion mode compared to the PP group (Figure 1A,B). Hierarchical clustering of metabolite heatmaps revealed clear separation between PP and PR, indicating a stable and pronounced effect of rearing system on the muscle metabolic profile. Specifically, positive ion mode analysis confirmed that PP displayed elevated expression of lipid and fatty acid metabolites (e.g., 2-linoleoyl glycerol, linoleoyl ethanolamide, ACar series). By comparison, the PR group was enriched in amino acid derivatives (e.g., N-acetyl-L-glutamic acid, N-acetyl-aspartic acid) and energy metabolism intermediates (e.g., glucose 6-phosphate, creatine phosphate, acetyl phosphate) (Figure 1C). In negative ion mode, PP exhibited marked upregulation of lipid-associated metabolites, including LPE 19:0, LysoPE 18:0 and LPC 18:0. In contrast, individuals from PR showed relatively higher levels of certain amino acids and derivatives, including N-acetyl-L-aspartic acid, N-lactoyl-phenylalanine, Asp-Glu, and D-2-aminoadipic acid as well as carbohydrate-related metabolites such as D-(-)-fructose and D-erythrose 4-phosphate (Figure 1D).

### 3.5. Intestinal Microbiota Characteristics of P. fulvidraco

**Figure 2:**
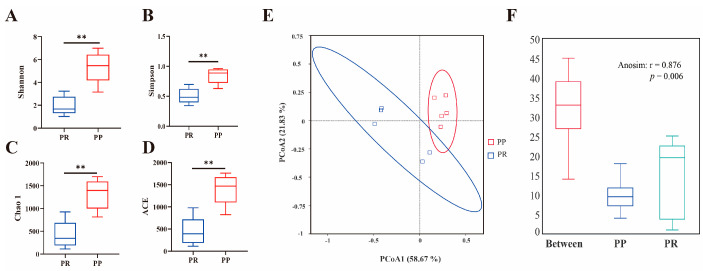
Differences in gut microbiota α- and β-diversity of yellow catfish between PP and PR groups. (**A**–**D**) show α-diversity indices for the two groups, including the Shannon index (**A**), Simpson index (**B**), Chao1 index (**C**) and ACE index (**D**); (**E**) shows principal coordinate analysis (PCoA) based on gut microbial community composition; and (**F**) presents ANOSIM results used to test differences in gut microbiota structure between the two groups. ** *p* < 0.01.

Intestinal microbial diversity differed significantly between the two groups. Compared with the rice-field culture group (PR), the pond culture group (PP) showed significantly higher Shannon, Simpson, Chao1 and ACE indices (*p* < 0.01), indicating greater gut microbial richness and diversity (Figure 2A–D). A total of 2478 OTUs were identified across both groups, with 461 OTUs shared between them, accounting for approximately 18.60% of the total detected OTUs. Principal coordinate analysis (PCoA) further revealed a clear separation between PP and PR communities, with PCoA1 and PCoA2 explaining 58.67% and 21.83% of the total variation, respectively (Figure 2E). This pattern was supported by ANOSIM analysis, which confirmed a significant difference in gut microbiota structure between the two groups (r = 0.876, *p* = 0.006) (Figure 2F). These results indicate that culture mode strongly shapes the composition and diversity of the gut microbiota in yellow catfish.

**Figure 3:**
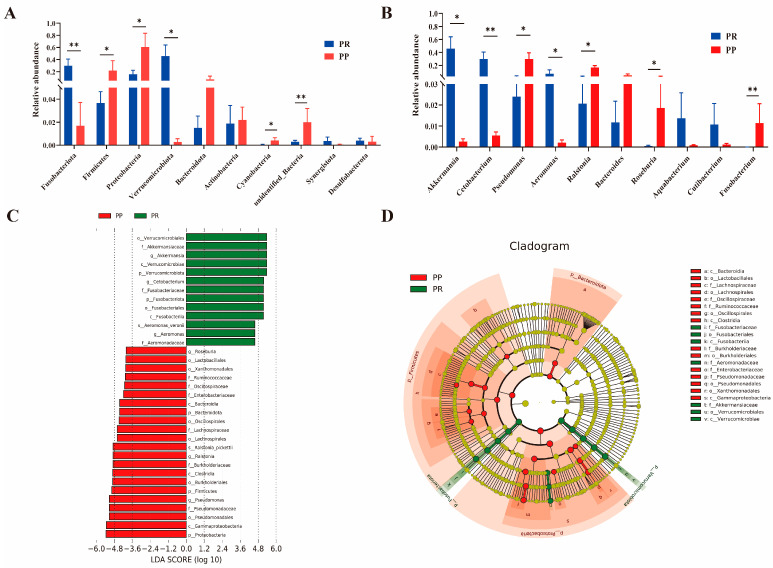
Taxonomic differences in the gut microbiota of yellow catfish between PP and PR groups. (**A**,**B**) show the mean relative abundances of dominant gut bacterial taxa within the whole microbial community at the phylum and genus levels, respectively. (**C**) represents differentially enriched taxa identified by LEfSe analysis and their corresponding LDA scores. (**D**) presents the LEfSe cladogram, illustrating discriminative taxa enriched in each group across taxonomic levels from phylum to genus. * *p* < 0.05 and ** *p* < 0.01.

At the phylum level, the gut microbial composition of yellow catfish differed markedly between the PP and PR groups. The percentages reported here represent the mean relative abundances of taxa within the whole gut microbial community at each taxonomic rank. Fusobacteriota (29.67%) and Verrucomicrobiota (45.62%) showed higher relative abundances in PR, whereas Firmicutes (21.61%) and Proteobacteria (60.69%) were enriched in PP (Figure 3A). At the genus level, PR was mainly characterized by higher abundances of *Akkermansia* (45.61%) and *Cetobacterium* (29.68%), while *Pseudomonas* (29.64%) and *Ralstonia* (16.67%) were more abundant in PP (Figure 3B). However, because the present data were generated from V3–V4 16S rRNA amplicons, species-level identification was not performed. LEfSe analysis further confirmed distinct culture mode-associated microbial signatures. The PP group was significantly enriched in Proteobacteria, Gammaproteobacteria, Pseudomonadales, Pseudomonadaceae, *Pseudomonas* and Firmicutes-related taxa, whereas the PR group was primarily enriched in Verrucomicrobiota, *Akkermansia* and Fusobacteriota-related taxa (Figure 3C,D). Together, these results indicate that culture mode substantially reshapes the dominant and discriminative gut bacterial taxa of yellow catfish. Tax4Fun analysis revealed that the level 2 KEGG pathway showed that the PR group was significantly enriched in gut microbial functions related to carbohydrate metabolism (*p* = 0.004), translation (*p* = 0.001) and energy metabolism (*p* = 0.006). In contrast, the PP group exhibited higher abundances of functions associated with environmental response, regulation and motility, including signal transduction (*p* < 0.001), enzyme families (*p* = 0.038) and transcription (*p* < 0.001) (Figure S3).

## 4. Discussion

Integrated rice–fish farming is increasingly recognized as a sustainable aquaculture model that can enhance resource use, ecosystem stability and product quality [1,3]. Although rice-field culture can reshape fish growth, nutrient deposition, metabolism and gut microbiota, these responses are species-specific [20,25,26]. Here, integrated analyses of growth traits, muscle nutrition, metabolomics and intestinal microbiota showed that pond culture (PP) and rice-field culture (PR) produced distinct physiological and metabolic phenotypes in yellow catfish. PR fish showed higher levels of several essential amino acids and amino acid-related metabolites, whereas PP fish accumulated more fatty acids and lipid-associated metabolites. These variations were accompanied by changes in gut microbial composition and predicted functions, indicating that the culture system modulates muscle quality through coordinated effects on nutrient deposition, host metabolism and intestinal microbial ecology.

### 4.1. Effects of Different Culture Systems on Growth Performance of P. fulvidraco

Growth performance is one of the most important indicators for evaluating the suitability of aquaculture systems. In the present study, body weight and body length did not differ significantly between PP and PR individuals, indicating that rice-field culture did not compromise the overall growth of yellow catfish under the experimental conditions. However, PP individuals exhibited significantly greater body width and body height than PR fish. We speculated that the greater body breadth and height of PP fish suggest that pond-cultured individuals had a more robust and fuller body shape, whereas rice-field-cultured fish tended to exhibit a relatively slimmer or more streamlined morphology. These differences may reflect culture-mode-related changes in body condition and energy allocation rather than differences in overall growth [27]. Similarly, a study on tilapia reported pronounced morphological differences among culture environments, including variation in body depth and head morphology and suggested that water flow, stocking density, culture system, temperature and genetic background may all contribute to morphological variation in farmed fish [28]. Pond environments generally provide a relatively stable feeding regime and lower spatial complexity, allowing fish to allocate more energy toward tissue deposition and body thickening. In contrast, rice-field systems contain shallow planting areas, refuge trenches and heterogeneous microhabitats that may increase swimming activity and energy expenditure [29,30]. Previous studies on rice–fish co-culture have similarly reported leaner body morphology and reduced visceral fat accumulation in fish reared in paddy ecosystems [9,31]. Therefore, the morphological differences observed in this study may reflect distinct strategies of energy utilization rather than differences in growth potential.

### 4.2. Effects of Culture Systems on Muscle Amino Acid Composition of P. fulvidraco

Muscle amino acid composition is a key determinant of nutritional value and sensory quality in fish products. In the present study, PR individuals exhibited significantly higher concentrations of Ser, Val, Ile and total essential amino acids (EAAs) than PP fish. Among these amino acids, Val and Ile belong to the branched-chain amino acid (BCAA) family, which plays critical roles in protein synthesis, muscle growth and energy metabolism. Increased BCAA levels are also considered beneficial for the nutritional quality of aquatic products. Previous fish nutrition studies have shown that appropriate dietary levels of valine can promote growth, improve protein utilization and activate TOR/S6K1-mediated protein synthesis signaling, whereas isoleucine may enhance muscle protein synthesis through the AKT-TOR-S6K1 pathway [32,33]. The enhancement of essential amino acids in PR fish is consistent with findings reported in several rice–fish co-culture studies [5,12]. For example, rice-field-cultured tilapia showed significantly higher levels of essential amino acids and flavor-related amino acids than pond-cultured fish [34,35]. Some studies have shown that selected essential amino acids also exert immunomodulatory and antioxidant functions. For example, valine and isoleucine can modulate key signaling pathways to enhance antioxidant enzyme activities and reduce oxidative damage, thereby contributing to improved physiological condition [36,37]. These improvements might have been attributed to the diverse natural food resources available in paddy ecosystems, including phytoplankton, benthic organisms, aquatic insects and detrital organic matter [38].

The metabolomic results further supported the amino acid enrichment observed in PR fish. Several amino acid-related metabolites, including N-acetyl-L-glutamic acid, N-acetyl-L-aspartic acid, Asp-Glu and N-lactoyl-phenylalanine, were significantly elevated in the PR group. N-acetyl-L-glutamic acid is an important regulator of nitrogen metabolism and amino acid biosynthesis, whereas N-acetyl-L-aspartic acid participates in amino acid turnover and cellular energy metabolism [39,40]. The enrichment of these metabolites suggests that rice-field culture may stimulate amino acid synthesis, transformation and utilization pathways. Published research has similarly demonstrated that ecological aquaculture systems can enhance amino acid metabolism and muscle nutrient deposition. For example, integrated rice–crayfish systems have been shown to affect amino acid-related metabolic pathways and improve or reshape muscle nutritional quality compared with conventional pond culture [41,42]. Likewise, fish reared under semi-natural or more physically demanding conditions may exhibit enhanced dietary protein utilization and altered amino acid/protein metabolism, reflecting increased locomotor activity and metabolic adaptation to heterogeneous environments [43,44]. Therefore, the higher amino acid content observed in PR individuals may result from the combined effects of diversified dietary inputs, altered energy allocation and enhanced amino acid metabolic activity.

### 4.3. Effects of Culture Systems on Muscle Fatty Acid Composition of P. fulvidraco

Unlike amino acids, fatty acid accumulation was significantly greater in individuals captured from ponds than in paddy fields. In the present study, pond-cultured yellow catfish exhibited higher concentrations of C14:0, C16:0, C18:0, C16:1, C18:2n6c, C20:4n6, C20:3n3, total saturated fatty acids (SFAs), monounsaturated fatty acids (MUFAs), n-3 polyunsaturated fatty acids (PUFAs) and total fatty acids. These results indicate that pond culture promoted lipid deposition and fatty acid accumulation in muscle tissue [11]. Furthermore, studies have found that pond-cultured fish often exhibit higher crude lipid content and greater fatty acid accumulation than fish reared in rice-field systems [5,45]. The relatively stable feeding conditions and lower activity levels in ponds may facilitate energy storage and lipid deposition [45]. In contrast, fish cultured in rice fields generally experience greater environmental complexity and locomotor activity, which can increase energy expenditure and reduce lipid accumulation [46,47]. However, the effects of rice-field culture on fatty acid composition are not always consistent across species. Previous studies on tilapia and carp have reported increased levels of DHA, EPA and n-3 PUFA in rice-field-cultured fish [10,13]. These improvements might be attributed to the consumption of natural food organisms rich in long-chain polyunsaturated fatty acids [48].

Our results showed the overall lower total fatty acid accumulation in PR individuals, suggesting that yellow catfish may respond differently to rice-field environments. Species-specific feeding ecology and lipid metabolism likely contribute to these contrasting outcomes. The metabolomic data provided additional evidence supporting enhanced lipid metabolism in pond-cultured fish. Several lipid-associated metabolites, including 2-linoleoyl glycerol, linoleoyl ethanolamide, acylcarnitines, LPC 18:0, LysoPE 18:0 and LPE 19:0, were significantly enriched in the PP group. Acylcarnitines are involved in mitochondrial fatty acid transport and β-oxidation, whereas lysophospholipids participate in membrane remodeling and lipid signaling [49,50,51]. Similar increases in lipid-derived metabolites have been reported in fish exhibiting elevated lipid deposition and active fatty acid metabolism [10,52,53]. Therefore, the combined fatty acid and metabolomic results indicate that pond culture promoted both lipid accumulation and lipid metabolic activity in yellow catfish muscle.

### 4.4. Metabolomic Responses to Different Culture Modes

Untargeted metabolomics revealed a clear separation between PP and PR groups, indicating that culture systems profoundly influenced muscle metabolic status. Compared with conventional nutritional analyses, metabolomics provides a more comprehensive understanding of the biochemical pathways underlying flesh quality formation [16,54]. The metabolic profile of PP individuals was characterized primarily by enrichment of lipid-related metabolites, which is consistent with the higher fatty acid content observed in muscle tissue. Similar metabolomic signatures have been reported in fish exposed to high-energy diets or intensive culture systems, where lipid synthesis and phospholipid turnover pathways are strongly activated [55,56]. On the other hand, fish from PR groups exhibited elevated levels of metabolites associated with amino acid metabolism, glycolysis and energy metabolism, including glucose-6-phosphate, creatine phosphate, acetyl phosphate, D-fructose and D-erythrose-4-phosphate. Glucose-6-phosphate is a central intermediate linking glycolysis, glycogen metabolism and the pentose phosphate pathway, whereas creatine phosphate functions as an important cellular energy reservoir [57]. An earlier study in carp demonstrated that muscle types differ markedly in glycolytic and energy-metabolic capacity under sustained swimming and locomotor demand [58]. In this context, the enrichment of energy-related metabolites in rice-field-cultured yellow catfish may reflect greater activity-driven metabolic turnover, as the structurally heterogeneous rice-field habitat likely promotes continuous movement, foraging and nutrient transformation.

Comparable findings have been reported in recent multi-omics studies of ecological aquaculture systems. For example, rice-field-cultured fish and crustaceans can exhibit enhanced or reprogrammed amino acid metabolism, carbohydrate utilization and oxidative energy-related pathways compared with conventionally cultured animals [6,59]. These metabolic adaptations are generally interpreted as responses to more complex environmental conditions and diversified food resources. Importantly, the metabolomic results bridge the gap between nutrient composition and physiological regulation. In this study, we found that the enrichment of amino acid-related metabolites in PR fish corresponded closely with the increased essential amino acid content, whereas the enrichment of lipid-derived metabolites in PP fish matched the observed fatty acid accumulation. Together, our findings indicate that culture mode influences flesh quality through systematic metabolic reprogramming rather than simple changes in nutrient intake alone.

### 4.5. Intestinal Microbiota Responses to Different Culture Modes

The intestinal microbiota plays a central role in nutrient digestion, energy metabolism, immune regulation and host adaptation [60]. In the present study, significant differences in intestinal microbial diversity and community composition were observed between two culture modes. Unexpectedly, PP individuals exhibited significantly higher Shannon, Simpson, Chao1 and ACE indices than PR fish, indicating greater microbial richness and diversity. This result differs from several previous studies reporting increased microbial diversity in rice-field-cultured fish [6,21]. For example, fish reared in rice-field systems are often exposed to a broader range of environmental microbes and natural food resources, which may promote a more diverse intestinal microbiota [6]. However, other studies suggested that ecological aquaculture systems can also reduce microbial diversity when selected beneficial taxa become dominant, leading to a more specialized but potentially functionally stable community [61]. Therefore, microbial diversity alone does not necessarily indicate a healthier or more functional microbial community.

At the phylum level, PR groups were enriched in Verrucomicrobiota and Fusobacteriota, whereas PP groups contained higher abundances of Proteobacteria and Firmicutes. At the genus level, *Akkermansia* and *Cetobacterium* dominated the PR group, while *Pseudomonas* and *Ralstonia* were more abundant in PP fish. *Akkermansia* is widely recognized as a mucin-degrading bacterium associated with intestinal barrier maintenance, metabolic regulation and host health in mammals and aquatic animals [62]. Several studies have linked increased *Akkermansia* abundance with improved nutrient utilization and metabolic homeostasis [63,64]. However, the present V3–V4 16S rRNA data provide only genus-level information and do not confirm species identity or functional traits. In the present study, *Akkermansia*-assigned reads accounted for 45.61% of the total bacterial community in rice-field-cultured yellow catfish. Although direct reports of high *Akkermansia* abundance in rice-field-cultured fish remain limited, previous studies have shown that integrated rice–aquatic animal systems can strongly reshape host-associated microbiota and may enrich specific beneficial or niche-associated taxa, including *Akkermansia* in rice–crayfish systems [65]. Compared with conventional ponds, rice-field systems provide a more heterogeneous habitat, including shallow rice-planting areas, refuge canals, natural food organisms, detrital inputs and sediment-associated microbial sources. These conditions may alter fish foraging behavior, energy expenditure, dietary substrate composition and gut environmental conditions [8]. Such ecological filtering could favor a more specialized intestinal microbial community, as also reflected by the lower microbial richness and diversity observed in the PR group [20]. Similarly, *Cetobacterium* is one of the most common beneficial bacteria in freshwater fish and has been associated with vitamin B12 synthesis, carbohydrate fermentation and amino acid metabolism [66]. The enrichment of *Akkermansia* and *Cetobacterium* in PR individuals may therefore contribute to the enhanced amino acid metabolism observed in this study.

*Pseudomonas* and *Ralstonia* showed higher relative abundances in the gut microbiota of pond-cultured yellow catfish. Both *Pseudomonas* and *Ralstonia* are metabolically versatile Proteobacteria that commonly occur in aquatic environments, including pond water, sediments, feed-associated microbial communities and fish intestines [67]. Functionally, members of *Pseudomonas* are metabolically versatile bacteria capable of utilizing diverse carbon and organic substrates, and several fish-related studies suggest that they may contribute to intestinal proteolysis, protein utilization, and host cellular homeostasis under specific culture or dietary conditions [68]. However, this genus also includes opportunistic pathogens that may cause infection under stress or immunosuppressed conditions [69]. Similarly, increased *Ralstonia* abundance has been observed in the gut microbiota of fish following pathogen challenge, suggesting that this genus may, in some contexts, be associated with microbial disturbance or environmental stress [70]. Thus, the enrichment of *Pseudomonas* and *Ralstonia* in pond-cultured yellow catfish may indicate greater microbial metabolic plasticity and sensitivity to environmental variation but may also imply a potential risk of opportunistic bacterial expansion if host immune homeostasis is disrupted.

Functional prediction analysis was broadly consistent with the taxonomic patterns while remaining an inference based on 16S rRNA gene profiles. The PR group showed higher predicted capacities for carbohydrate metabolism, translation and energy metabolism, whereas PP fish were enriched in functions related to signal transduction, transcription, motility and xenobiotic biodegradation. Integrating these patterns with the metabolomic data suggests a working model in which rice-field culture may favor taxa such as *Akkermansia* and *Cetobacterium*, enhance microbial carbohydrate and energy metabolism, and thereby support amino acid biosynthesis and nutrient utilization by the host. By contrast, pond culture may select for microbial communities with greater environmental responsiveness and lipid-associated metabolic potential, contributing to increased lipid deposition in muscle. Although these associations remain correlative, they provide a useful framework for understanding how cultural environments may shape flesh quality through host-microbiota interactions.

Beyond these biological associations, the present findings have practical relevance for quality-oriented aquaculture. Compared with previous studies that mainly focused on environmental performance, rice growth, stocking density or individual nutritional traits in rice–fish systems, this study integrates muscle amino acid and fatty acid composition, untargeted metabolomics and intestinal microbiota in a direct comparison between pond- and rice-field-cultured yellow catfish. This integrated approach shows that different culture modes do not simply improve or reduce fish quality uniformly but instead shape distinct quality-forming pathways. From an applied perspective, these findings may support ecological aquaculture management, quality-oriented production and product differentiation. Pond culture may favor body fullness and fatty acid deposition, whereas rice-field culture maintained overall growth while producing a leaner phenotype and higher levels of selected amino acids. These culture-mode-specific traits provide useful information for production planning, quality labeling, market positioning and consumer-oriented evaluation of yellow catfish products. However, the study was limited to one experimental base and one culture cycle, with relatively small pooled sample sizes. The effects of different feeding regimes were not evaluated, and the 16S rRNA data provide only relative abundance and genus-level information, with functional predictions remaining inferential. Future studies with larger sample sizes, multiple sites and seasons, varied feeding conditions and complementary validation methods such as metagenomics, targeted qPCR, absolute microbial quantification and sensory evaluation are needed to confirm the broader applicability and mechanisms of these findings.

## 5. Conclusions

This study shows that rice-field and pond culture systems shape distinct growth, nutritional, metabolic and microbial phenotypes in yellow catfish. Under comparable stocking and feeding conditions, rice-field culture did not compromise overall growth but produced a leaner phenotype with higher levels of selected amino acids together with amino acid and energy-related metabolites. By contrast, pond culture promoted greater body thickening and muscle fatty acid deposition, accompanied by enrichment of lipid-associated metabolites, indicating enhanced lipid accumulation and metabolic activity. The gut microbiota also displayed clear culture mode-specific patterns. Rice-field-cultured yellow catfish were characterized by higher abundances of *Akkermansia* and *Cetobacterium*, together with greater predicted capacities for carbohydrate metabolism, translation and energy metabolism, whereas pond-cultured fish harbored higher abundances of *Pseudomonas* and *Ralstonia*, as well as functions associated with environmental responsiveness and motility. These findings suggest that rice-field and pond culture systems do not uniformly improve or reduce yellow catfish quality but instead drive distinct quality-forming pathways. By integrating nutritional, metabolomic and microbial evidence, this study provides a framework for optimizing rice–yellow catfish co-culture toward quality-oriented aquaculture.

## Figures and Tables

**Table 1:** The hydrolyzed amino acid composition in the muscle of *Pelteobagrus fulvidraco* under different farming conditions (*n* = 3).

Amino Acids (g/100 g, ww)	Groups		
	PP	PR	Sig.
Aspartic acid (ASP)	1.75 ± 0.04	1.88 ± 0.03	0.053
Threonine (Thr)	0.78 ± 0.02	0.84 ± 0.01	0.062
Serine (Ser)	0.68 ± 0.01	0.72 ± 0.01	0.022 *
Glutamic acid (Glu)	2.49 ± 0.07	2.63 ± 0.06	0.216
Glycine (Gly)	0.86 ± 0.03	0.82 ± 0.02	0.313
Alanine (Ala)	0.93 ± 0.06	0.90 ± 0.07	0.730
Cysteine (Cys)	0.12 ± 0.01	0.12 ± 0.01	0.795
Valine (Val)	0.75 ± 0.01	0.81 ± 0.01	0.009 **
Methionine (Met)	0.37 ± 0.03	0.33 ± 0.02	0.357
Isoleucine (Ile)	0.64 ± 0.01	0.74 ± 0.02	0.003 **
Leucine (Leu)	1.30 ± 0.01	1.35 ± 0.02	0.051
Tyrosine (Tyr)	0.51 ± 0.02	0.52 ± 0.01	0.887
Phenylalanine (Phe)	0.57 ± 0.03	0.58 ± 0.02	0.922
Lysine (Lys)	1.52 ± 0.02	1.61 ± 0.04	0.099
Histidine (His)	0.31 ± 0.02	0.31 ± 0.01	0.791
Arginine (Arg)	0.85 ± 0.01	0.87 ± 0.01	0.184
Proline (Pro)	0.54 ± 0.06	0.53 ± 0.02	0.838
ΣEAA	7.10 ± 0.03	7.44 ± 0.10	0.027 *
ΣNEAA	7.88 ± 0.13	8.11 ± 0.13	0.105
ΣTAA	14.99 ± 0.12	15.55 ± 0.22	0.247

EAA: Essential amino acid, NEAA: Non-essential amino acid, TAA: Total amino acid, ww: wet weight. All values are expressed as the mean ± SEM (*n* = 3). * *p* < 0.05 and ** *p* < 0.01 represent significant statistical difference.

**Table 2:** Hydrolyzed fatty acid composition in muscle of *P. fulvidraco* in different farming conditions.

Fatty Acids (mg/100 g)	Groups		
	PP	PR	Sig.
C14:0	13.93 ± 1.24	7.13 ± 1.09	0.015 *
C16:0	420.00 ± 23.11	263.30 ± 30.26	0.015 *
C18:0	143.47 ± 6.91	104.47 ± 11.68	0.013 *
SFA	577.40 ± 30.48	374.90 ± 43.04	0.045 *
C16:1	58.97 ± 7.28	23.93 ± 3.92	0.045 *
C18:1n9c	800.13 ± 82.46	464.30 ± 81.97	0.153
C20:1	33.00 ± 2.01	21.37 ± 3.52	0.280
C22:1n9	9.20 ± 3.05	11.83 ± 1.63	0.046 *
MUFA	901.30 ± 89.40	521.43 ± 88.08	0.018 *
C20:2	32.20 ± 1.35	23.43 ± 1.81	0.231
C18:3n3	36.17 ± 4.01	28.13 ± 5.02	0.206
C20:5n3	6.10 ± 0.32	3.87 ± 0.16	0.603
C22:6n3	75.87 ± 8.05	57.30 ± 5.59	0.489
C18:2n6c	547.97 ± 48.10	410.90 ± 61.28	0.004 **
C20:3n6	42.07 ± 3.12	36.43 ± 2.49	0.131
C20:4n6	23.90 ± 3.11	21.80 ± 2.05	0.018 *
C20:3n3	5.37 ± 0.53	4.10 ± 0.41	0.039 *
PUFA	733.47 ± 45.21	556.47 ± 59.71	0.077
n-3 PUFA	123.50 ± 6.63	92.03 ± 1.61	0.010 *
n-6 PUFA	613.93 ± 45.94	469.13 ± 61.96	0.134
n-3: n-6 ratio	0.20 ± 0.016	0.20 ± 0.026	0.996
Total FA	2248.33 ± 164.66	1480.93 ± 195.65	0.040 *

SFA, saturated fatty acid; MUFA, monounsaturated fatty acid; PUFA, polyunsaturated fatty acid. All values are expressed as the mean ± SEM (*n* = 3). * *p* < 0.05 and ** *p* < 0.01 represent significant statistical difference.

## Data Availability

The original contributions presented in the study are included in the article/Supplementary Material, further inquiries can be directed to the corresponding authors.

## Supplementary Materials

The following supporting information can be downloaded at: https://www.mdpi.com/article/10.3390/foods15152666/s1, Figure S1: PCA score plot assessing quality of metabolomics data; Figure S2: Classification and pathway annotation overview for small-molecule metabolites; Figure S3: Comparative analysis of predicted gut microbiota functions in *P. fulvidraco* under different culture modes; Table S1: Growth performance of *P. fulvidraco*; Table S2: The free amino acid composition in muscle of *P. fulvidraco* under different farming models.

## Abbreviations

The following abbreviations are used in this manuscript:

PP	Pond culture/pond-cultured group
PR	Rice-field culture/rice-field-cultured group
LC–MS/MS	Liquid chromatography–tandem mass spectrometry
Ser	Serine
Val	Valine
Ile	Isoleucine
n-3/ω-3	Omega-3 fatty acids
n-6/ω-6	Omega-6 fatty acids
DHA	Docosahexaenoic acid
PUFA/PUFAs	Polyunsaturated fatty acid(s)
MS-222	Tricaine methanesulfonate
FAMEs	Fatty acid methyl esters
UHPLC	Ultra-high-performance liquid chromatography
KEGG	Kyoto Encyclopedia of Genes and Genomes
LIPID MAPS	Lipid Metabolites and Pathways Strategy
PCA	Principal component analysis
PLS-DA	Partial least squares discriminant analysis
SILVA	SILVA ribosomal RNA gene database
QIIME	Quantitative Insights Into Microbial Ecology
LDA	Linear discriminant analysis
LEfSe	Linear discriminant analysis effect size
EAA	Essential amino acid(s)
NEAA	Non-essential amino acid(s)

## References

[1] Xie J., Hu L., Tang J., Wu X., Li N., Yuan Y., Yang H., Zhang J., Luo S., Chen X. (2011). Ecological mechanisms underlying the sustainability of the agricultural heritage rice-fish coculture system. Proc. Natl. Acad. Sci. USA.

[2] Hou Y., Yu Z., Jia R., Li B., Zhu J. (2024). Integrated rice-yellow catfish farming resulting in variations in the agricultural environment, rice growth performance, and soil bacterial communities. Environ. Sci. Pollut. Res..

[3] Hu L., Zhang J., Ren W., Guo L., Cheng Y., Li J., Li K., Zhu Z., Zhang J., Luo S. (2016). Can the co-cultivation of rice and fish help sustain rice production?. Sci. Rep..

[4] Feng J., Li F., Zhou X., Xu C., Fang F. (2016). Nutrient removal ability and economical benefit of a rice-fish co-culture system in aquaculture pond. Ecol. Eng..

[5] Yan T., Xie Y.-Y., Zhou B., Kuang X., Li Q.-Z., Zhao F.-Q., Li Q.-D., He B. (2024). Rice-Fish Farming Improved Antioxidant Defences, Glucose Metabolism, and Muscle Nutrient of *Carassius auratus* in Sichuan Province. Metabolites.

[6] Wang E., Zhou Y., Liang Y., Ling F., Xue X., He X., Zhai X., Xue Y., Zhou C., Tang G. (2022). Rice flowering improves the muscle nutrient, intestinal microbiota diversity, and liver metabolism profiles of tilapia (*Oreochromis niloticus*) in rice-fish symbiosis. Microbiome.

[7] Diao W., Jia R., Hou Y., Dong Y., Li B., Zhu J. (2023). Effects of Stocking Density on the Growth Performance, Physiological Parameters, Antioxidant Status and Lipid Metabolism of *Pelteobagrus fulvidraco* in the Integrated Rice-Fish Farming System. Animals.

[8] Cheng R., Ying Z., Yang Y., Zhang C., Zhou W., Zhang Z., Ding H., Zhou Y., Zhang C. (2025). Changes of intestinal microbiota and liver metabolomics in yellow catfish (*Pelteobagrus fulvidraco*) before and after rice flowering in rice-fish symbiosis farmed mode. Front. Microbiol..

[9] Zhao Y., Zhou W., Li M., Zhang Y., Lv W., Huang W., Yang H., Yuan Q., Li M. (2025). Effects of Different Farming Models on Muscle Quality, Intestinal Microbiota Diversity, and Liver Metabolism of Rice Field Eel (*Monopterus albus*). Foods.

[10] Rong Y., Li B., Hou Y., Zhang L., Jia R., Zhu J. (2024). Influences of Stocking Density on Antioxidant Status, Nutrients Composition, and Lipid Metabolism in the Muscles of *Cyprinus carpio* under Rice-Fish Co-Culture. Antioxidants.

[11] Zhou F., Bu W., Fan H., Guo S., Qi M., Yao G., Bei Y., Huang Y., Zhu S., Ding X. (2024). A Comparative Study on the Muscle and Gut Microbiota of *Opsariichthys bidens* from Rice Field and Pond Culture Breeding Modes. Metabolites.

[12] He J., Feng P., Lv C., Lv M., Ruan Z., Yang H., Ma H., Wang R. (2020). Effect of a fish–rice co-culture system on the growth performance and muscle quality of tilapia (*Oreochromis niloticus*). Aquac. Rep..

[13] Zhu Y., Ji Y., Zhou X., He X., Xue X., Zhang J., Tang H., Zhou Y., Zhang C. (2024). Rice-fish symbiosis improves the muscle nutrition and intestinalflora diversity of tilapia. Isr. J. Aquac. BAMID.

[14] Yang C., Liu K., Deng Y., Wang Q., Cao S., Zhou Q. (2025). Multi-Omics Analysis Provides New Insights into the Interplay Between Gut Microbiota, Fatty Acid Metabolism, and Immune Response in Cultured and Wild *Coilia nasus* from the Yangtze River Area in China. Microorganisms.

[15] Sidira M., Agriopoulou S., Smaoui S., Varzakas T. (2024). Omics-Integrated Approach (Metabolomics, Proteomics and Lipidomics) to Assess the Quality Control of Aquatic and Seafood Products. Appl. Sci..

[16] Li X., Wang S., Zhang M., Jiang H., Qian Y., Wang R., Li M. (2023). Comprehensive analysis of metabolomics on flesh quality of yellow catfish (*Pelteobagrus fulvidraco*) fed plant-based protein diet. Front. Nutr..

[17] Zhang L., Zhao Z., Xiong D., Fang W., Li B., Fan Q., Yang K., Wang X. (2011). Effects of ration level on growth, nitrogenous excretion and energy budget of juvenile yellow catfish, *Pelteobagrus fulvidraco* (Richardson). Aquac. Res..

[18] Zhou Y., Xiong Y., He X., Xue X., Tang G., Mei J. (2023). Depuration and Starvation Regulate Metabolism and Improve Flesh Quality of Yellow Catfish (*Pelteobagrus fulvidraco*). Metabolites.

[19] Yang S., Du J., Duan Y.-L., Xiao Q., Li N.-Q., Lin Q., Zhao L.-L., Du Z.-J., Zhou J., Du J. (2018). Differences in the digestive enzyme activity, intestinal mucosa and microbial community in loach cultivated in two separate environments. BMC Microbiol..

[20] Tao L., Chai J., Liu H., Huang W., Zou Y., Wu M., Peng B., Wang Q., Tang K.A.-O. (2022). Characterization and Dynamics of the Gut Microbiota in Rice Fishes at Different Developmental Stages in Rice-Fish Coculture Systems. Microorganisms.

[21] Yang S., Duan Y., Zhang J., Zhou J., Liu Y., Du J., Zhao L., Du Z., Han S. (2017). Observational comparisons of intestinal microbiota characterizations, immune enzyme activities, and muscle amino acid compositions of loach in paddy fields and ponds in Sichuan Province. Appl. Microbiol. Biotechnol..

[22] Want E.J., Masson P., Michopoulos F., Wilson I.D., Theodoridis G., Plumb R.S., Shockcor J., Loftus N., Holmes E., Nicholson J.K. (2013). Global metabolic profiling of animal and human tissues via UPLC-MS. Nat. Protoc..

[23] Edgar R.C. (2013). UPARSE: Highly accurate OTU sequences from microbial amplicon reads. Nat. Methods.

[24] Quast C., Pruesse E., Yilmaz P., Gerken J., Schweer T., Yarza P., Peplies J., Glockner F.O. (2013). The SILVA ribosomal RNA gene database project: Improved data processing and web-based tools. Nucleic Acids Res..

[25] Guan W., Li K., Li K. (2022). Bacterial communities in co-cultured fish intestines and rice field soil irrigated with aquaculture wastewater. AMB Express.

[26] Fan H., Ye J., Zhou F., Su W., Wang X., Bu W., Bei Y., Chen J., Guo S., Zheng B. (2026). Comparative evaluation of nutritional quality and flavor characteristics for *Acrossocheilus fasciatus* muscle in different aquaculture systems. Food Chem..

[27] Fenkes M., Shiels H.A., Nudds R.L. (2018). Body shape and robustness response to water flow during development of brown trout *Salmo trutta* parr. J. Fish. Biol..

[28] Lopez A.M., Arias C.M., Morales A.T., Angel M.O., Betancur J. (2019). Body shape variation between farms of tilapia (*Oreochromis* sp.) in Colombian Andes using landmark based geometric morphometrics. Lat. Am. J. Aquat. Res..

[29] Halwart M., Borlinghaus M., Kaule G. (1996). Activity pattern of fish in rice fields. Aquaculture.

[30] Claireaux G., Lefrançois C. (2007). Linking environmental variability and fish performance: Integration through the concept of scope for activity. Philos. Trans. R. Soc. B.

[31] Karapanagiotidis I.T., Yakupitiyage A., Little D.C., Bell M.V., Mente E. (2010). The nutritional value of lipids in various tropical aquatic animals from rice–fish farming systems in northeast Thailand. J. Food Compos. Anal..

[32] Jiang Q., Yan M., Zhao Y., Zhou X., Yin L., Feng L., Liu Y., Jiang W., Wu P., Wang Y. (2021). Dietary isoleucine improved flesh quality, muscle antioxidant capacity, and muscle growth associated with AKT/TOR/S6K1 and AKT/FOXO3a signaling in hybrid bagrid catfish (*Pelteobagrus vachelli*♀ × *Leiocassis longirostris*♂). J. Anim. Sci. Biotechnol..

[33] Ahmad I., Ahmed I., Dar N.A. (2021). Dietary valine improved growth, immunity, enzymatic activities and expression of TOR signaling cascade genes in rainbow trout, *Oncorhynchus mykiss* fingerlings. Sci. Rep..

[34] Gong Y., Luo M., Zhang X., Qin N., Zhu W., Wang L., Fu J., Dong Z. (2025). Evaluation of muscle quality and movement-related traits in two common carp strains under a rice–fish farming system. Aquac. Int..

[35] Inayat M., Abbas F., Hafeez-Ur-Rehman M., Mahmud A.J.P.O. (2023). Optimizing rice-fish co-culture: Investigating the impact of rice spacing density on biochemical profiles and production of genetically modified tilapia (*Oreochromis* spp.) and *Cyprinus carpio*. PLoS ONE.

[36] Andersen S.M., Waagbø R., Espe M. (2016). Functional amino acids in fish nutrition, health and welfare. Front. Biosci..

[37] Habte-Tsion H.M. (2020). A review on fish immuno-nutritional response to indispensable amino acids in relation to TOR, NF-κB and Nrf2 signaling pathways: Trends and prospects. Comp. Biochem. Physiol. B Biochem. Mol. Biol..

[38] Mortillaro J.-M., Dabbadie L., Raminoharisoa A.E., Paradis A., Martel P., Andriamarolaza R., Raliniaina M., Mikolasek O., Aubin J. (2022). Trophic functioning of integrated rice-fish farming in Madagascar: Insights from stable isotopes (δ13C & δ15N). Aquaculture.

[39] Moffett J.R., Ross B., Arun P., Madhavarao C.N., Namboodiri A.M. (2007). N-Acetylaspartate in the CNS: From neurodiagnostics to neurobiology. Prog. Neurobiol..

[40] Shi D., Allewell N.M., Tuchman M. (2015). The N-Acetylglutamate Synthase Family: Structures, Function and Mechanisms. Int. J. Mol. Sci..

[41] Liu T., Tian J., Zhang L., Chen J., Yu Y., Tian C., Gan J. (2026). The Effects of Different Culture Modes on the Nutritional Quality of *Procambarus clarkii* and Mechanistic Insights: A Metabolomic Perspective. Biology.

[42] Wang S., Zhang S., Luo L., Zhang R., Guo K., Su J., Zhao Z. (2025). Biological Characteristics and Chemical Composition of Crayfish (*Procambarus clarkii*) Reared in Two Different Culture Modes in Cold Regions of China. Foods.

[43] Marhuenda-Egea F.C., Sanchez-Jerez P. (2025). Metabolomic Insights into Wild and Farmed Gilthead Seabream (*Sparus aurata*): Lipid Composition, Freshness Indicators, and Environmental Adaptations. Molecules.

[44] Beltrán M., Fernández-Borrás J., Médale F., Pérez-Sánchez J., Kaushik S., Blasco J. (2009). Natural abundance of 15N and 13C in fish tissues and the use of stable isotopes as dietary protein tracers in rainbow trout and gilthead sea bream. Aquac. Nutr..

[45] Zhang X., Wang J., Tang R., He X., Li L., Takagi Y., Li D. (2019). Improvement of Muscle Quality of Grass Carp (*Ctenopharyngodon idellus*) with a Bio-Floating Bed in Culture Ponds. Front. Physiol..

[46] Burns A., Gatlin D.M. (2022). Effects of sustained swimming exercise on growth and body composition responses of Nile tilapia (*Oreochromis niloticus*), red drum (*Sciaenops ocellatus*), and hybrid striped bass (*Morone chrysops* × *M. saxatilis*). Fish. Physiol. Biochem..

[47] Simpkins D.G., Hubert W.A., del Martinez Rio C., Rule D.C. (2003). Effect of Swimming Activity on Relative Weight and Body Composition of Juvenile Rainbow Trout. N. Am. J. Fish. Manag..

[48] Hixson S., Sharma B., Kainz M., Wacker A., Arts M.T.J.E.R. (2015). Production, distribution, and abundance of long-chain omega-3 polyunsaturated fatty acids: A fundamental dichotomy between freshwater and terrestrial ecosystems. Environ. Rev..

[49] Longo N., Frigeni M., Pasquali M. (2016). Carnitine transport and fatty acid oxidation. Biochim. Biophys. Acta.

[50] Zheng L., Lin Y., Lu S., Zhang J., Bogdanov M. (2016). Biogenesis, transport and remodeling of lysophospholipids in Gram-negative bacteria. BBA-Mol. Cell Biol. Lipids.

[51] Law S.H., Chan M.L., Marathe G.K., Parveen F., Chen C.H., Ke L.Y. (2019). An Updated Review of Lysophosphatidylcholine Metabolism in Human Diseases. Int. J. Mol. Sci..

[52] Xu H., Luo X., Wei Y., Liang M. (2022). Dietary lysophosphatidylcholine regulates diacylglycerol, cardiolipin and free fatty acid contents in the fillet of turbot. Food Chem. X.

[53] Hao M., Yi L., Cheng W., Zhu J., Zhao S. (2024). Lipidomics analysis reveals new insights into crisp grass carp associated with meat texture. Heliyon.

[54] Honghao Z., Jasmine C., Rong T., Li L., Jianguo X., Dapeng L.J.G. (2018). Metabolomics investigation of dietary effects on flesh quality in grass carp (*Ctenopharyngodon idellus*). GigaScience.

[55] Roldi G., Robath S., Porto C., Pilau E., Giaquinto P., Neto J.F., Lala B., Mari G.C., Santos C.T., Anastácio G.N. (2025). Stocking density modulates tilapia (*Oreochromis niloticus*) performance and fillet quality through phospholipid remodeling. Sci. Rep..

[56] Chen J., Zhuo M., Jiang J., Yu A., Yu D., Huang F. (2023). Effects of dietary lipid levels on fiber quality, lipidomic profiles, antioxidant and inflammation responses in muscle of yellow catfish *Pelteobagrus fulvidraco*. Aquac. Rep..

[57] Wuertz S., Reiser S. (2023). Creatine: A valuable supplement in aquafeeds?. Rev. Aquac..

[58] Johnston I.A., Davison W., Goldspink G. (1977). Energy metabolism of carp swimming muscles. J. Comp. Physiol. A.

[59] Jia R., Dong Y., Hou Y., Feng W., Li B., Zhu J. (2023). Transcriptome Analysis Reveals the Effect of Stocking Density on Energy Metabolism in the Gills of *Cherax quadricarinatus* under Rice-Crayfish Co-Culture. Int. J. Mol. Sci..

[60] Butt R.L., Volkoff H. (2019). Gut Microbiota and Energy Homeostasis in Fish. Front. Endocrinol..

[61] Yan Q., Li J., Yu Y., Wang J., He Z., Van Nostrand J.D., Kempher M.L., Wu L., Wang Y., Liao L. (2016). Environmental filtering decreases with fish development for the assembly of gut microbiota. Environ. Microbiol..

[62] Cani P.D., Depommier C., Derrien M., Everard A., de Vos W.M. (2022). Akkermansia muciniphila: Paradigm for next-generation beneficial microorganisms. Nat. Rev. Gastroenterol. Hepatol..

[63] Huang Z., Wang Y., Feng S., Zhang Y., Zhang X., Chang X., Yang G., Meng X. (2024). Effects of pasteurized *Akkermansia muciniphila* on lipid metabolism disorders induced by high-fat diet in zebrafish (*Danio rerio*). Aquac. Rep..

[64] Everard A., Belzer C., Geurts L., Ouwerkerk J.P., Druart C., Bindels L.B., Guiot Y., Derrien M., Muccioli G.G., Delzenne N.M. (2013). Cross-talk between *Akkermansia muciniphila* and intestinal epithelium controls diet-induced obesity. Proc. Natl. Acad. Sci. USA.

[65] Chen L., Xu J., Wan W., Xu Z., Hu R., Zhang Y., Zheng J., Gu Z. (2022). The Microbiome Structure of a Rice-Crayfish Integrated Breeding Model and Its Association with Crayfish Growth and Water Quality. Microbiol. Spectr..

[66] Zhang Z., Fan Z., Yi M., Liu Z., Ke X., Gao F., Cao J., Wang M., Chen G., Lu M. (2022). Characterization of the core gut microbiota of Nile tilapia (*Oreochromis niloticus*): Indication of a putative novel Cetobacterium species and analysis of its potential function on nutrition. Arch. Microbiol..

[67] Liu G., Mao C., Li Q., Huo D., Li T. (2025). Comparative genomic analysis reveals the adaptive traits of *Ralstonia* spp. in aquatic environments. Front. Microbiol..

[68] Song C., Wen H., Liu G., Ma X., Lv G., Wu N., Chen J., Xue M., Li H., Xu P. (2022). Gut Microbes Reveal Pseudomonas Medicates Ingestion Preference via Protein Utilization and Cellular Homeostasis Under Feed Domestication in Freshwater Drum, *Aplodinotus grunniens*. Front. Microbiol..

[69] Oh W.T., Kim J.H., Jun J.W., Giri S.S., Yun S., Kim H.J., Kim S.G., Kim S.W., Han S.J., Kwon J. (2019). Genetic Characterization and Pathological Analysis of a Novel Bacterial Pathogen, *Pseudomonas tructae*, in Rainbow Trout (*Oncorhynchus mykiss*). Microorganisms.

[70] Kim D.-G., Lee S.-J., Lee J.M., Lee E.-W., Jang W.J. (2023). Changes in the Gut Microbiota Composition of Juvenile Olive Flounder (*Paralichthys olivaceus*) Caused by Pathogenic Bacterial Infection. Fishes.

