# Supplementary material for: Comparative Analysis of Muscle Nutrients, Lipid Metabolism and Intestinal Microbiota in Pelteobagrus fulvidraco from Rice Field and Pond Culture Modes
Source: Foods. 2026 Jul 29;15(15):2666. doi: 10.3390/foods15152666 (PMC13464755; doi:10.3390/foods15152666)
Supplement: Supplementary file 1 [file foods-15-02666-s001.zip › foods-4421835-supplementary.pdf]

Table S1. Growth performance of *P. fulvidraco*

	PP	PR
Body weight (g)	181.69 ± 5.04	185.91 ± 6.23
Body length (cm)	22.26 ±0.20	22.40 ± 0.32
Body breadth (cm)	4.05 ± 0.05	3.82 ± 0.05
Body height (cm)	4.85 ± 0.06	4.47 ± 0.07

Values are presented as means ± SEM. PP, pond group. PR, rice-fish group.

Table S2. The free amino acid composition in muscle of *P. fulvidraco* under different farming models

Amino acids (g/100g, ww)	Groups		
	Pond-farming	Rice-farming	Sig.
Asp	3.67 ± 0.18	4.67 ±0.44	0.103
Thr	15.00 ± 1.00	12.33 ± 0.33	0.065
Ser	6.30 ± 0.32	5.83 ± 0.07	0.228
Glu	7.63 ± 0.79	9.63 ± 0.23	0.074
Gly	64.33 ± 2.03	71.33 ± 2.85	0.116
**Ala**	**14.67 ± 2.03**	**27.33 ± 0.67**	**0.004****
Cys	1.02 ± 0.04	0.85 ± 0.06	0.063
**Val**	**4.67 ± 0.24**	**7.17 ± 0.29**	**0.003****
**Ile**	**3.97 ± 0.03**	**6.40 ± 0.25**	**0.010***
Leu	13.67 ± 0.67	14.67 ± 0.66	0.349
Lys	9.83 ± 0.60	10.33 ± 0.33	0.507
His	5.13 ± 0.27	5.23 ± 0.18	0.774
**Delicate amino acids (ASP+Glu)**	**11.30 ± 0.75**	**14.30 ± 0.59**	**0.034***
**Sweet amino acids (Gly+Ala+Ser+Thr+Lys)**	**110.13 ± 3.17**	**127.17 ± 2.89**	**0.017***
**TFAA**	**149.89 ± 4.75**	**175.78 ± 2.69**	**0.009****

Results were expressed as mean ±SE. TFAA, total of free amino acid. *$p < 0.05$ and **$p < 0.01$ represent significant statistical difference.

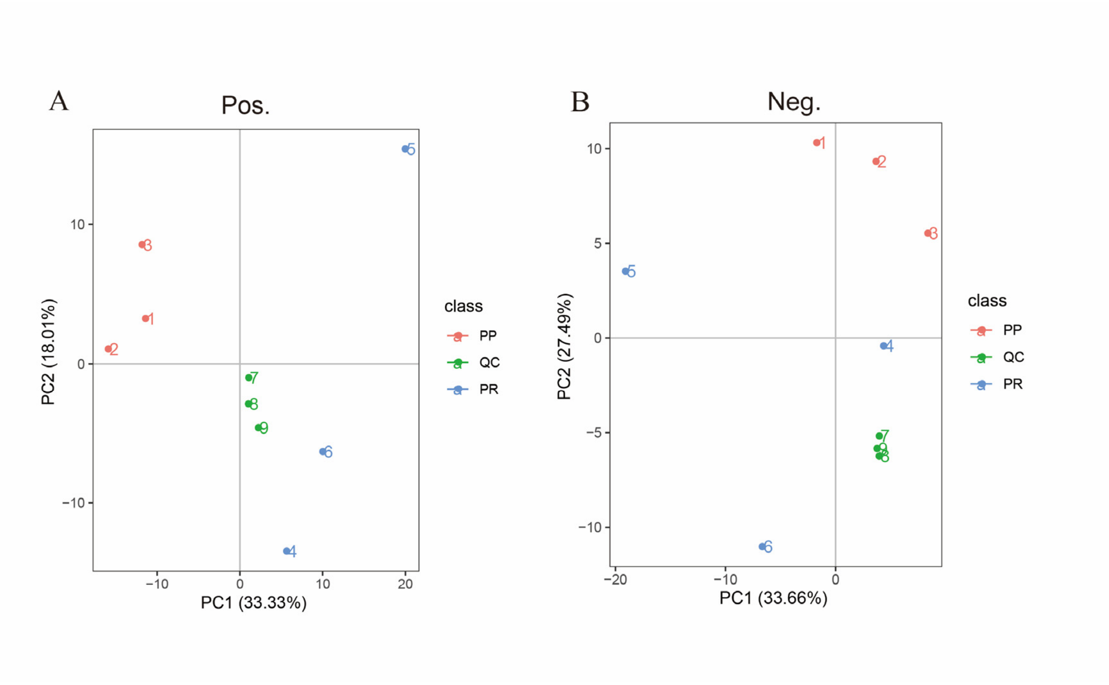

Figure S1. PCA score plot assessing quality of metabolomics data. (**A, B**) PCA score plot in positive (Pos) and negative (Neg) ion modes, respectively. PP, pond cultured group; PR, rice-fish cocultured group; QC, quality control samples. Tight clustering of quality control (QC) samples indicates better method stability and higher data quality.

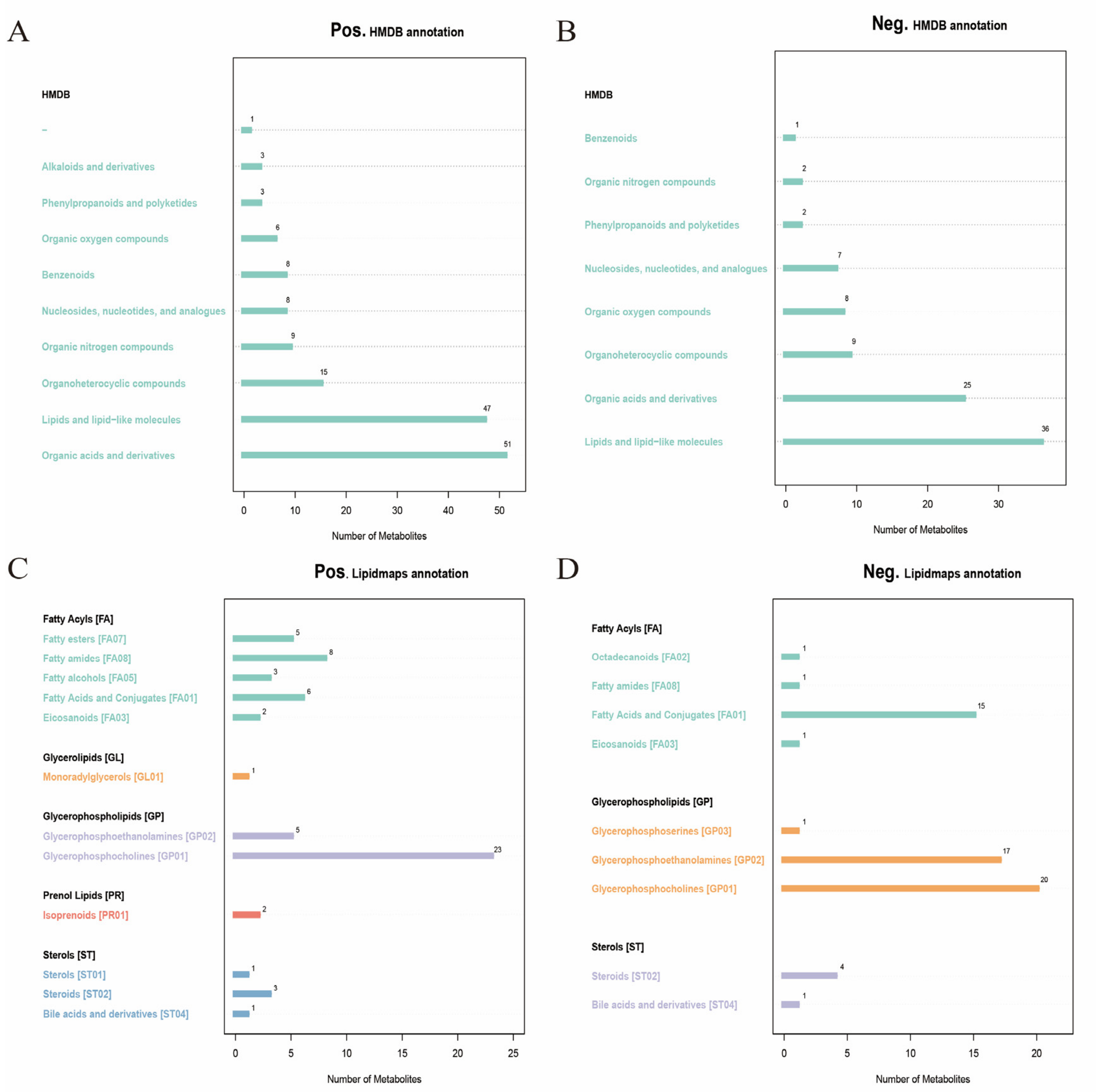

Figure S2. Classification and pathway annotation overview for small-molecule metabolites. (**A, B**) Classification of metabolites annotated to the Human Metabolome Database (HMDB) in positive (Pos) and negative (Neg) ion modes. (**C, D**) Classification of metabolites annotated to the LIPID MAPS database in positive (Pos) and negative (Neg) ion modes.

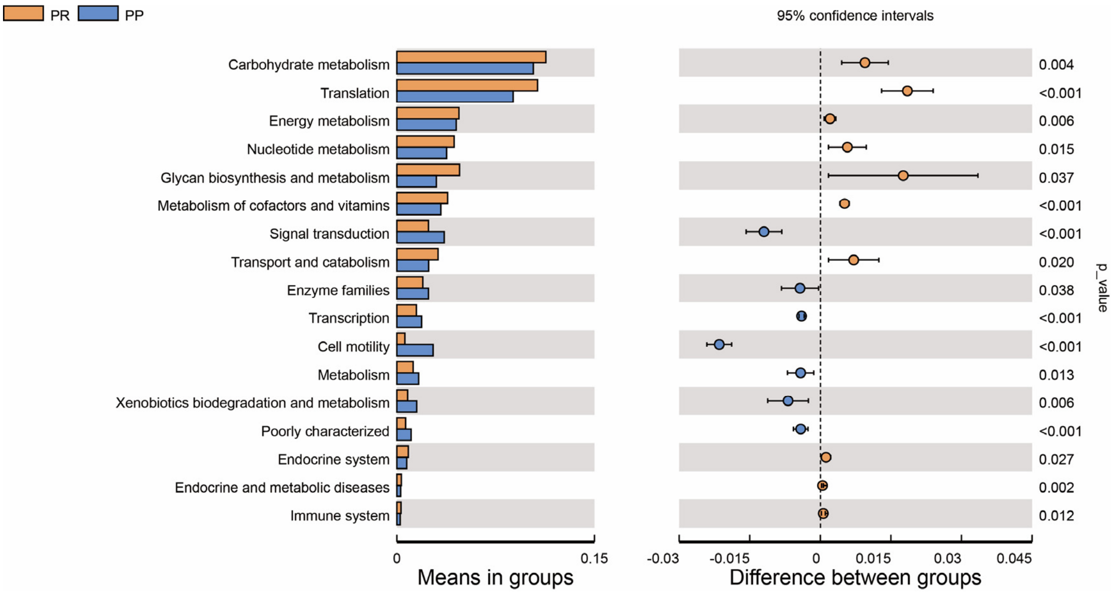

Figure S3. Comparative analysis of predicted gut microbiota functions in *P. fulvidraco* under different culture modes. Functional potential of the gut microbiota in PP and PR groups was inferred from 16S rRNA sequencing data and compared at KEGG level 2. The bar plot on the left shows the mean relative abundance of each functional category in the two groups. Points and error bars on the right indicate between-group differences and 95% confidence intervals; the dashed vertical line denotes no difference between groups. $p < 0.05$ was considered statistically significant.
